# Catamenial Pneumothorax Without Thoracic Endometriosis: A Case Report From the Gulf Cooperation Council (GCC) Region

**DOI:** 10.7759/cureus.94888

**Published:** 2025-10-18

**Authors:** Ahmed Ahmed, Nissar Shaikh, Firdos Ummunnisa, Umm E Amara, Umm E Nashrah

**Affiliations:** 1 Anesthesia, Hamad Medical Corporation, Doha, QAT; 2 Critical Care Medicine, Hamad Medical Corporation, Doha, QAT; 3 Obstetrics and Gynecology, Halima Al-Tamimi OBGY Clinic, Doha, QAT; 4 Critical Care, Deccan College of Medical Sciences, Hyderabad, IND

**Keywords:** catamenial pneumothorax, endometriosis, laparoscopy, menstrual cycle, vats

## Abstract

Catamenial pneumothorax is a rare clinical condition first described over seven decades ago. It typically occurs within 72 hours before or after menstruation and is most frequently observed in women of reproductive age. Reports from the Gulf Cooperation Council (GCC) region, including Qatar, remain limited, and underreporting has also been noted in developed countries such as Australia. This report presents a case of catamenial pneumothorax without evidence of thoracic abnormal endometriosis. A 37-year-old woman presented repeatedly to the emergency department with chest pain occurring during menstruation. Chest radiography showed a right-sided pneumothorax. Video-assisted thoracoscopy revealed right upper lobe blebs, which were excised. Laparoscopy identified the spread of endometrial tissue in the pelvis, ovaries, and uterus. The patient was treated with hormonal oral contraceptive therapy and remains under outpatient follow-up. Catamenial pneumothorax can occur in the absence of endometrial thoracic tissue. A high index of suspicion is essential for diagnosis in reproductive-aged women with recurrent spontaneous pneumothorax.

## Introduction

Catamenial pneumothorax is the occurrence of spontaneous pneumothorax within 72 hours of menstrual onset, potentially extending up to 96 hours [1]. Although first described more than seven decades ago, recent advances in diagnostic modalities have led to an increase in reported cases [2]. However, catamenial pneumothorax remains largely underdiagnosed or misdiagnosed, making its true incidence challenging to establish. Catamenial pneumothorax is underreported in the Gulf Cooperation Council (GCC) region, including Qatar. A case of pleural endometriosis presenting as cyclical chest pain has been reported in Doha, Qatar [3]. Even in developed countries such as Australia, catamenial pneumothorax remains underreported and underrecognized [4]. To our knowledge, no prior case of catamenial pneumothorax without evidence of thoracic endometrial tissue involvement has been documented in Qatar. Therefore, this report aims to investigate a case of recurrent spontaneous pneumothorax in a female patient, consistent with catamenial pneumothorax.

## Case presentation

A 37-year-old female patient, with an incidental detection of porphyria in urine, presented repeatedly to the emergency department with right-sided pleuritic chest pain radiating to the neck, back, and shoulder. The pain was associated with mild dyspnea during each menstrual cycle over the past year. No fever, cough, palpitations, or weight loss were reported. On examination, she was afebrile (37°C), with a heart rate of 86 beats/min, blood pressure of 124/80 mmHg, respiratory rate of 20 breaths/min, and SpO₂ of 99% on 2 L/min oxygen. She appeared stable but anxious. Cardiovascular and abdominal examinations were unremarkable, and breath sounds were decreased on the right side without added sounds. Neurological examination was normal.

**Figure 1:**
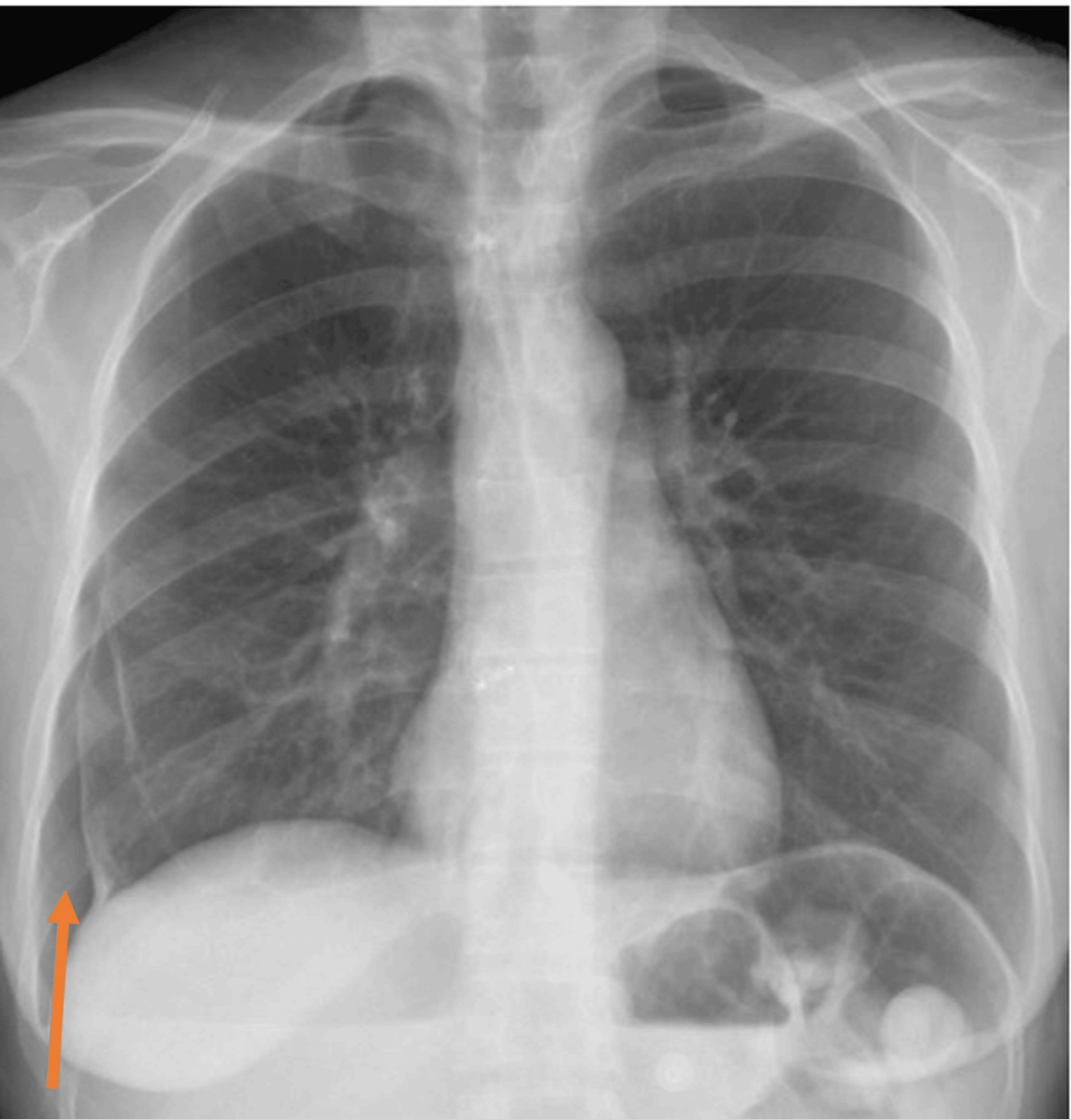
Chest radiograph Chest radiograph demonstrating a right-sided pneumothorax with no focal lung lesions or infiltrates. The absence of underlying lung pathology supports a spontaneous etiology

Her history was significant for recurrent right-sided pneumothorax. She underwent video-assisted thoracoscopic surgery (VATS) with bleb excision in March 2013 following an initial episode. In 2018, she experienced multiple emergency department visits for pleuritic chest pain. A computed tomography scan of the thorax during one of these visits revealed a right pneumothorax, which was managed conservatively. In February 2019, she re-presented with chest pain, and chest radiography revealed a small right pneumothorax. On arrival, she was vitally stable and not in distress. Chest radiography showed a small right-sided pneumothorax (Figure 1). Initial management was conservative, followed by VAT, which revealed some abnormal bullae in the right upper lobe, which were excised, and the remaining examination appeared normal. No pleural or diaphragmatic biopsies were taken during this procedure, as there was no macroscopic evidence of endometrial implants or diaphragmatic fenestrations. However, the possibility of occult or microscopic thoracic endometriosis, or undetected diaphragmatic microperforations, cannot be completely excluded and may account for the cyclical pattern of pneumothorax observed in this patient.. Laboratory studies show hemoglobin of 11.8 g/dL with otherwise normal hematological and biochemical parameters. A right-sided chest drain was inserted, resulting in lung re-expansion.

**Figure 2:**
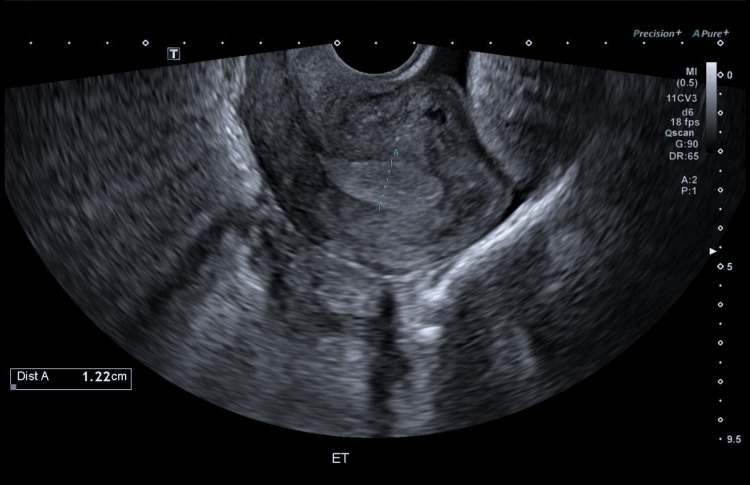
Pelvic ultrasonography Pelvic ultrasonography showing a retroverted uterus and increased endometrial thickness, suggestive of endometrial hyperplasia or endometriosis

Subsequent VATS again demonstrated abnormal bullae in the right upper lobe, which were excised, with no evidence of diaphragmatic involvement or intrathoracic endometrial implants. Preoperative pelvic ultrasonography revealed a retroverted uterus with increased endometrial thickness (Figure 2).

**Figure 3:**
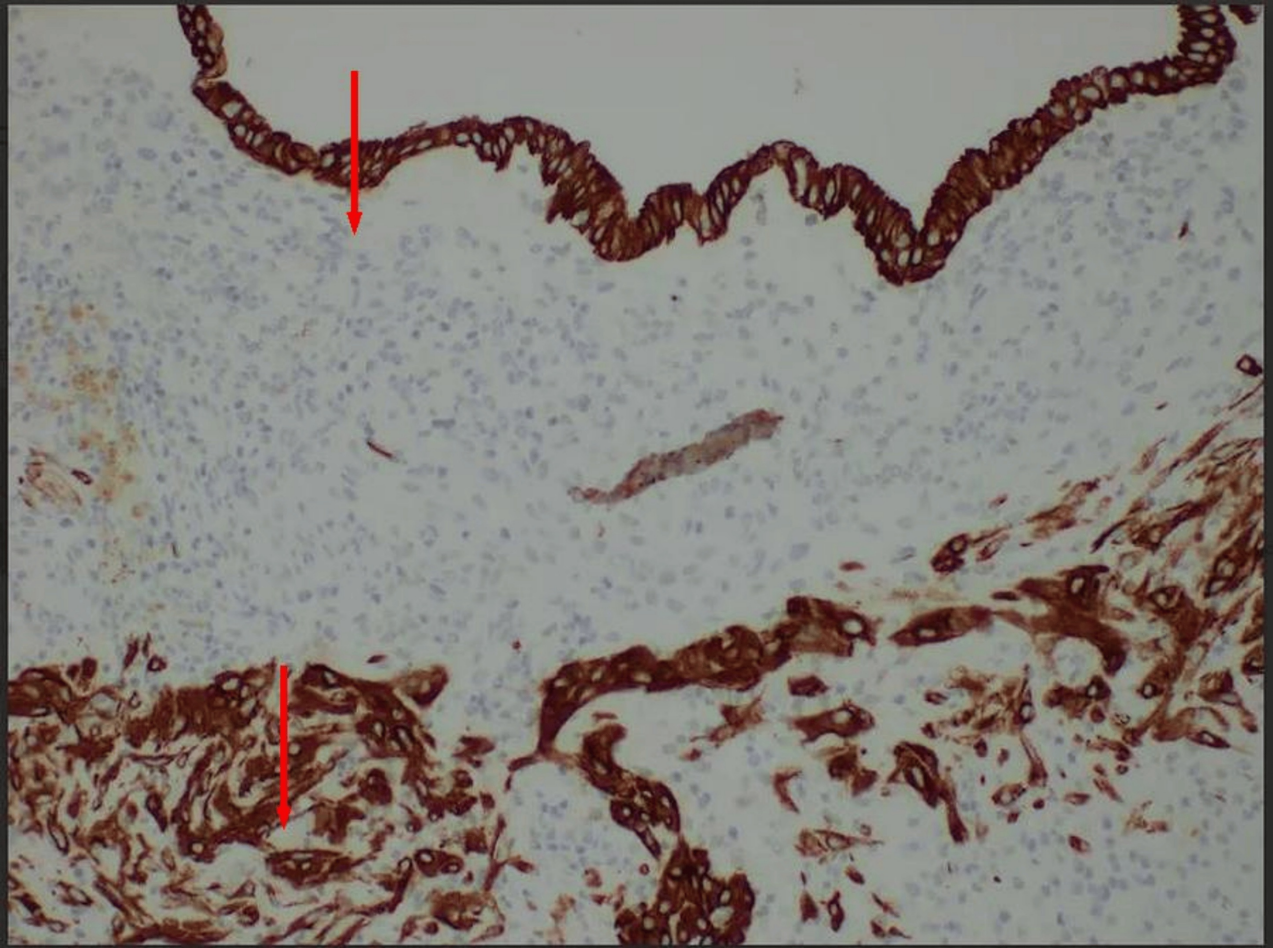
Punch biopsy specimen collected from posterior uterine serosa and right pelvic sidewall showing endometrial tissue The endometrial glands (top) and stroma (bottom) show strong CD10 immunopositivity, confirming the presence of ectopic endometrial tissue

Diagnostic laparoscopy showed multiple endometriotic implants involving the bilateral pelvic sidewalls, posterior uterine serosa, bilateral ovaries and ligaments, and the pouch of Douglas. Punch biopsies were obtained, and histopathological analysis confirmed ectopic endometrial glands and stroma, positive for CD10 (Figure 3).

Following consultation, the patient was initiated on a continuous combined oral contraceptive pill (ethinylestradiol 30 µg + gestodene 75 µg, 1 tablet orally once daily) as per Hamad General Hospital guidelines. Additional medication included amitriptyline 25 mg orally daily and tramadol 50 mg orally thrice daily as needed. These agents were initiated for chronic neuropathic pelvic pain that persisted despite prior intermittent nonsteroidal anti-inflammatory drug (NSAID) use. Management was coordinated with the institutional multidisciplinary pain team, which recommended the addition of low-dose amitriptyline for neuropathic modulation and tramadol for breakthrough pain control, while minimizing long-term NSAID exposure due to gastrointestinal intolerance and limited efficacy in this patient. Escalation to gonadotropin-releasing hormone (GnRH) agonist therapy (e.g., leuprolide acetate 3.75 mg intramuscularly every four weeks or goserelin 3.6 mg subcutaneously every 28 days) was discussed but not initiated because the patient opted against GnRH agonist therapy after counseling, citing concern over hypo-estrogenic side effects such as vasomotor symptoms and bone-density loss, and preferred to continue with combined oral contraceptive therapy, which she tolerated well and found effective. She was discharged with plans for outpatient follow-up.

## Discussion

Catamenial pneumothorax is defined as at least two episodes of spontaneous pneumothorax occurring consecutively with menstruation. This condition was initially reported in a patient who experienced 12 episodes within one year, each coinciding with her menstrual cycle [4]. The true incidence remains uncertain, as the condition is frequently underrecognized and misdiagnosed despite increasing awareness [5]. Proposed etiopathogenetic mechanisms include metastatic, hormonal, and anatomical theories. 

The hormonal physiology hypothesis suggests that elevated prostaglandin F2α induces vasoconstriction and bronchoconstriction, resulting in alveolar rupture and pneumothorax. Additionally, cyclical fluctuations in estrogen and progesterone levels are believed to increase vascular permeability and promote endometrial tissue responsiveness, potentially weakening pleural integrity and predisposing to alveolar rupture during menses. Pulmonary bullae or blebs may also rupture during hormonal fluctuations, leading to pneumothorax in the absence of pathological or endometrial lesions, as seen in several reported cases [6]. The metastatic theory proposes that endometrial tissue disseminates to the lungs through lymphatic or venous pathways, where subsequent necrosis of endometrial tissue precipitates pneumothorax [7].

The anatomical theory proposes that air enters the thoracic cavity through a transgenital-transdiaphragmatic pathway. During menstruation, the absence of cervical mucous allows air to ascend through the cervix and fallopian tubes into the peritoneal cavity, then through diaphragmatic defects, and migrate into the thorax, resulting in pneumothorax [8]. In the present case, no diaphragmatic fenestrations or defects were identified during thoracoscopy, although the possibility of microscopic or functionally patent microperforations cannot be entirely excluded.

Diagnosis of catamenial pneumothorax requires a high index of suspicion, particularly in reproductive-aged women with elevated CA-125 levels. Serum CA-125 may be mildly elevated in some patients with thoracic or pelvic endometriosis and can serve as a supportive but nonspecific biomarker; however, its diagnostic utility is limited because levels may remain normal or be influenced by other benign gynecologic conditions.

Clinical diagnosis can be supported by history and imaging studies, but definitive evaluation is best achieved through VATS and laparoscopy, which allow visualization and therapeutic excision of lesions [9,10]. Although catamenial pneumothorax often resolves spontaneously, it potentially progresses to a surgical emergency in cases of functional compromise or tension pneumothorax, necessitating intercostal tube drainage.

A combined approach integrating surgical excision and hormonal therapy is considered the most effective treatment strategy [11]. Surgical management involves excision of endometrial tissue from lungs, pleural repair, and closure of diaphragmatic defects, ideally performed during menstruation to optimize visualization of endometrial tissues [12]. Operating during menstruation enhances intraoperative identification of endometrial foci because lesions are more vascular and friable and may appear reddish-brown or bluish compared to the surrounding pleura. This timing also allows detection of active bleeding points or diaphragmatic fenestrations that may be obscured at other phases of the cycle, improving completeness of resection and reducing recurrence rates. Hormonal therapy options include oral contraceptives and GnRH agonists; oral contraceptives are generally preferred, as GnRH agonists induce hypoestrogenic effects, which may lead to treatment discontinuation and higher chances of Catamenial pneumothorax recurrence [9]. Recent studies also support the use of progestin-only agents, such as dienogest or norethindrone acetate, which effectively suppress ovulation while minimizing hypoestrogenic symptoms [13,14]. In selected cases, the levonorgestrel-releasing intrauterine system (LNG-IUS) provides continuous local progestogenic activity that limits endometrial proliferation and offers excellent tolerability and long-term adherence. These options may be particularly beneficial for patients who cannot tolerate estrogen-containing regimens or require sustained hormonal suppression [13,15].

## Conclusions

Catamenial pneumothorax is an uncommon and frequently underdiagnosed condition that may occur even in the absence of visible thoracic endometrial tissue. Early recognition and a coordinated surgical-hormonal treatment approach are key to preventing recurrence and improving outcomes. Greater regional awareness and multicenter collaboration are needed to establish standardized diagnostic pathways and optimize long-term management strategies.
